# Selective Symmetrical Encephalomalacia in a Maine Coon Crossbred Kitten in Italy

**DOI:** 10.3390/vetsci13090891

**Published:** 2026-08-31

**Authors:** Serena Frizziero, Cristina Casalone, Letizia Tripodi, Francesca Cimino, Tiziana Avanzato, Camilla Testori, Valerio Carta, Roberto Zoccola, Erika Messana, Carlo Cantile, Barbara Iulini

**Affiliations:** 1 Istituto Zooprofilattico Sperimentale del Piemonte, Liguria e Valle d’Aosta, Via Bologna 148, 10154 Turin, Italy; serena.frizziero@izsplv.it (S.F.); cristina.casalone@izsplv.it (C.C.); ltripodi@aslto4.piemonte.it (L.T.); francescaa.c@libero.it (F.C.); tiziana.avanzato@izsplv.it (T.A.); camilla.testori@izsplv.it (C.T.); valerio.carta@izsplv.it (V.C.); roberto.zoccola@izsplv.it (R.Z.); erika.messana@izsplv.it (E.M.); 2 Azienda Sanitaria Locale Torino 4, Servizio Veterinario Area C, Piazza Gino Bellandy 1, 10082 Cuorgnè, Italy; 3 Dipartimento di Scienze Veterinarie, Università di Pisa, Viale delle Piagge 2, 56124 Pisa, Italy; carlo.cantile@unipi.it

**Keywords:** brain diseases, neuropathological lesions, selective symmetrical encephalomalacia, spongiosis, vacuolation

## Abstract

Selective symmetrical encephalomalacia (SSE) is a rare neurological disorder characterized by bilateral and symmetrical areas of malacia within specific regions of the central nervous system and has been reported in humans and several animal species. This report describes a case of SSE in a six-month-old Maine Coon crossbred kitten from Italy that presented with central vestibular dysfunction, rapidly progressive neurological deterioration, proprioceptive deficits, respiratory impairment, seizures, and abnormal vocalization. Clinical evaluation, hematological and biochemical analyses, and ancillary diagnostic testing were performed to investigate the underlying cause and evaluate differential diagnoses. Despite supportive care, the animal died naturally a few days after the onset of clinical signs. A complete postmortem examination, including histopathological evaluation of the central nervous system, was subsequently performed. Neuropathological analysis revealed multifocal, bilateral, and symmetrical malacic and spongiform lesions affecting multiple brain regions, compatible with an SSE pattern. Comparison with previously reported cases in humans and dogs showed marked similarities in lesion distribution and morphology. This case expands the limited knowledge of SSE-like neuropathological lesions in cats and highlights the value of neuropathological examination in the diagnosis of rare neurological disorders presenting with rapidly progressive clinical signs.

## Simple Summary

Selective symmetrical encephalomalacia is a rare disease that causes damage to specific areas of the brain on both sides in a symmetrical pattern. It has been described in young animals and in humans, but only one case has been reported in cats. This report describes a six-month-old kitten in Italy that developed severe neurological problems, including loss of balance, difficulty controlling body movements, breathing problems, seizures, and abnormal vocalization. Despite clinical examinations and laboratory testing, no clear cause of the disease could be identified while the animal was alive. The kitten died a few days after the onset of clinical signs. After death, detailed examination of the brain revealed characteristic areas of tissue destruction affecting multiple regions of the central nervous system. These findings were consistent with selective symmetrical encephalomalacia. Additional diagnostic investigations helped rule out other diseases that can cause similar neurological signs. By comparing the lesions observed in this kitten with those previously reported in dogs and humans, this report expands current knowledge of this uncommon condition and highlights the importance of postmortem examination in investigating unexplained neurological diseases in young animals.

## 1. Introduction

Selective symmetrical encephalomalacia (SSE) encompasses a group of rare degenerative neurological disorders characterized by bilateral and symmetrical, well-defined areas of encephalomalacia in specific regions of the brain and spinal cord. The disease has been described in both humans and animals, particularly in young individuals. Histopathological lesions may range from spongiosis to extensive tissue cavitation and are typically associated with vascular proliferation, astrogliosis, perivascular mononuclear cuffing, and vacuolated astrocytes, whereas neurons are often relatively spared [1].

In veterinary medicine, several forms of SSE have been reported in different species and are frequently associated with inherited metabolic or mitochondrial dysfunction. In dogs, recognized syndromes include hereditary polioencephalomyelopathy of Australian Cattle Dogs, Alaskan Husky encephalopathy [2,3,4], subacute necrotizing encephalopathy in Yorkshire Terriers [5], and severe subacute necrotizing encephalopathy in American Staffordshire Bull Terriers [6]. Similar disorders have also been described in cattle, including Limousin [7] and Simmental calves—classified as multifocal subacute necrotizing encephalomyelopathy [8]—and Angus calves, in which multifocal symmetrical necrotizing encephalomyelopathy has been reported [9]. Neuropathological lesions in Limousin cattle are localized in the basal nuclei, brainstem, and spinal cord, with greater involvement of the white matter. In Simmental and Simmental-cross cattle, lesions are mainly observed in the olivary nuclei. In Angus calves, involvement of the parasympathetic vagal nucleus, lateral cuneate nucleus, and olivary nuclei at the level of the medulla oblongata has been described.

In humans, the condition most closely resembling SSE is Leigh syndrome, a severe neurodegenerative disorder of infancy associated with defects in mitochondrial energy metabolism and characterized by bilateral, symmetrical spongiform and malacic lesions preferentially localized in the basal ganglia, thalamus, cerebellum, and brainstem [5,10]. Comparable neuropathological features, including spongiform degeneration, vascular proliferation, demyelination, and relative neuronal preservation, have been documented in both human and veterinary cases [2,5,6,11,12]. Importantly, the Leigh-like/SSE neuropathological pattern is characterized not only by bilateral and symmetrical lesion distribution, but also by the combination of selective neuroanatomical involvement, neuropil spongiosis and malacia, vascular proliferation, gliosis, and, in some cases, relative preservation of neuronal cell bodies.

Despite the growing body of literature describing SSE-like disorders in humans, dogs, and cattle, reports in cats remain exceptionally rare. To date, only a single case has been described in a cat [13]. Consequently, the clinical presentation, pathological features, and differential diagnostic considerations of SSE in cats remain poorly characterized.

The present report describes a case of SSE in a six-month-old Maine Coon crossbred kitten presenting with rapidly progressive neurological disease. The aim of this report was to characterize the clinical and neuropathological findings, investigate potential differential diagnoses, and compare the observed lesions with those previously reported in other species. The findings contribute to the limited knowledge of SSE in cats and further support the occurrence of this uncommon neurodegenerative condition in cats.

## 2. Case Presentation

A six-month-old outdoor Maine Coon crossbred kitten was presented to a private veterinary clinic for evaluation of rapidly progressive neurological signs. At presentation, the animal exhibited central vestibular dysfunction characterized by ataxia, incoordination, disorientation, blindness, lethargy, and a variable appetite. Three days later, the clinical condition deteriorated further, with the onset of proprioceptive deficits, respiratory impairment, seizures, and abnormal vocalization. According to the owner, a trivalent vaccine had been administered two weeks before the onset of clinical signs. Despite supportive care, the animal died naturally.

Routine serum biochemistry and a complete blood count revealed no significant abnormalities. Blood gas analysis was within normal limits except for an increased serum lactate concentration (47.2 mg/dL; reference range, 5.4–22.5 mg/dL). The antibody test for feline immunodeficiency virus (FIV) and feline leukemia virus (FeLV) was negative, whereas a weakly positive result was obtained for feline coronavirus (FCoV). Parasitological examination of feces yielded negative results.

Following death, a complete necropsy was performed at the Istituto Zooprofilattico Sperimentale del Piemonte, Liguria e Valle d’Aosta. The brain was collected for histopathological, immunohistochemical, microbiological, and molecular investigations and sectioned along a paramedian plane. The larger portion was fixed in 10% buffered formaldehyde solution for histological examination, whereas the smaller portion was frozen at −20 °C for microbiological analyses. For histological evaluation, selected regions of the central nervous system, including the telencephalon, diencephalon, mesencephalon, pons, cerebellum, and obex, were routinely processed, embedded in paraffin, sectioned at 5 µm, and evaluated by light microscopy after hematoxylin and eosin (H & E) staining.

Immunohistochemistry (IHC) was performed on all brain sections using a monoclonal antibody directed against the feline coronavirus nucleocapsid protein (CCV2-2; 1:500 dilution; Santa Cruz Biotechnology Inc., Dallas, TX, USA). In addition, brainstem and cerebellar sections were tested for accumulation of the disease-associated prion protein (PrP^Sc^) using the monoclonal antibody L42 (1:250 dilution; R-Biopharm AG, Darmstadt, Germany). Selected lesioned brain areas were immunostained for glial fibrillary acidic protein (GFAP; 1:1000 dilution; polyclonal antibody; Dako, Hamburg, Germany) using the avidin–biotin–peroxidase complex (ABC) technique [14].

Molecular analyses were also performed. Polymerase chain reaction (PCR) was performed to detect FeLV in spleen, kidney, and brain, whereas *Toxoplasma gondii* was investigated in brain and muscle tissues. Nucleic acids were extracted using the All-Prep^®^ DNA/RNA Mini Kit (Qiagen, Hilden, Germany). DNA was used for *T. gondii* detection by real-time PCR targeting a fragment of the B1 gene [15], using specific primers and a TaqMan probe. FCoV RNA was investigated by RT-PCR targeting a fragment of the membrane (M) gene, using the assay described by Pratelli et al. [16], with modifications. Extracted RNA was reverse-transcribed and amplified using the OneStep RT-PCR Kit (Qiagen, Hilden, Germany) with specific primers. Amplification products were detected by electrophoresis on a 2% agarose gel, followed by GelRed™ (Biotium, Fremont, CA, USA) staining and ultraviolet (UV) transillumination.

Real-time RT-PCR for feline calicivirus (FCV), targeting open reading frame 1 (ORF1), and PCR for feline herpesvirus type 1 (FHV-1), targeting the thymidine kinase (TK) gene, were performed on lung tissue. For FCV detection, RNA was extracted from tissue according to the manufacturer’s instructions for TRI Reagent^®^ (Sigma-Aldrich, St. Louis, MO, USA; catalog no. T9424). The primers, probe, and PCR protocol targeting ORF1 were those described in [17]. For FHV-1 detection, DNA was extracted according to the manufacturer’s instructions for the ExtractMe Genomic DNA Kit (catalog no. EM13), and the primers and PCR protocol targeting the TK gene described in [18] were used.

Direct immunofluorescence (IF) on cell monolayers and virus isolation were performed for feline panleukopenia virus (FPV) using intestinal tissue. For FPV isolation, a 10% (*w*/*v*) intestinal homogenate was prepared in Eagle’s minimum essential medium (EMEM) containing 5× antibiotics and centrifuged to remove tissue debris. The sample was filtered through a 0.45-µm pore-size syringe filter, and 200 µL of the filtrate was inoculated onto actively growing CRFK (Crandell-Rees feline kidney) cells in 24-well plates containing EMEM supplemented with 10% (*v*/*v*) fetal bovine serum. Plates were incubated at 37 °C in 5% CO_2_ for 72 h. The cell culture supernatant was subsequently harvested after freeze–thaw cycles and used to inoculate fresh CRFK cells for a second passage. After the second passage, the culture supernatant was used to infect fresh CRFK cells grown on chamber slides for a third passage. After 72 h, infected cells were fixed in cold acetone for 20 min and subjected to direct immunofluorescence using an anti-FPV monoclonal antibody conjugated to fluorescein isothiocyanate (VMRD; catalog no. CJ-FPL-MAB-10ML), according to the manufacturer’s instructions.

**Figure 1:**
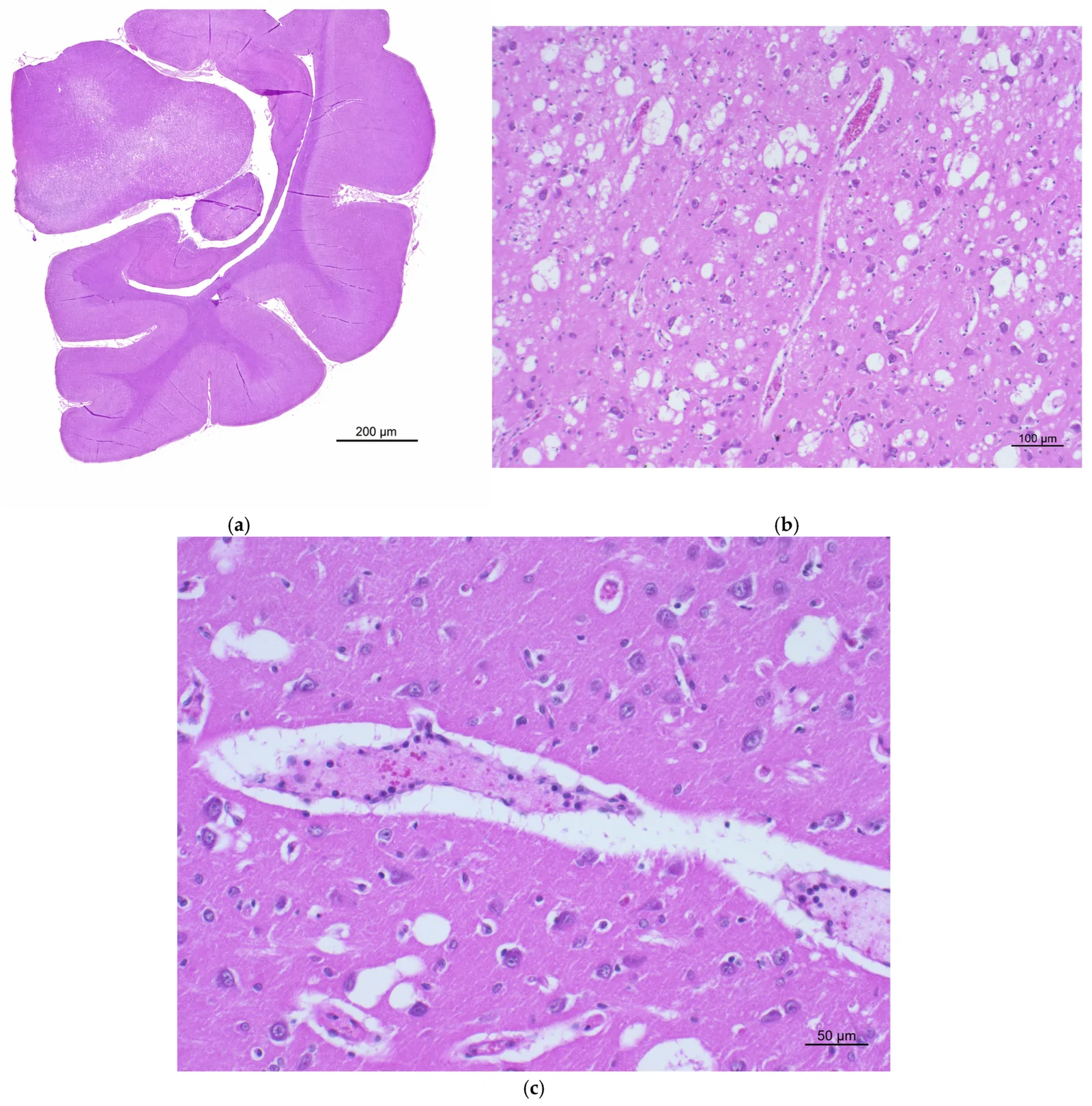
(**a**) Whole section at the level of the thalamus, hippocampus, and parieto-occipital cortex. Bilateral and symmetrical vacuolation in the thalamus. H & E; 2.5×. (**b**) Thalamus showing marked neuropil vacuolation and spongiosis, with relative preservation of neuronal cell bodies and prominent vascular profiles. H & E; 10×. (**c**) Thalamus showing focal perivascular lymphomonocytic cuffing within a dilated perivascular space, with relative preservation of surrounding neuronal cell bodies. H & E; 20×.

**Figure 2:**
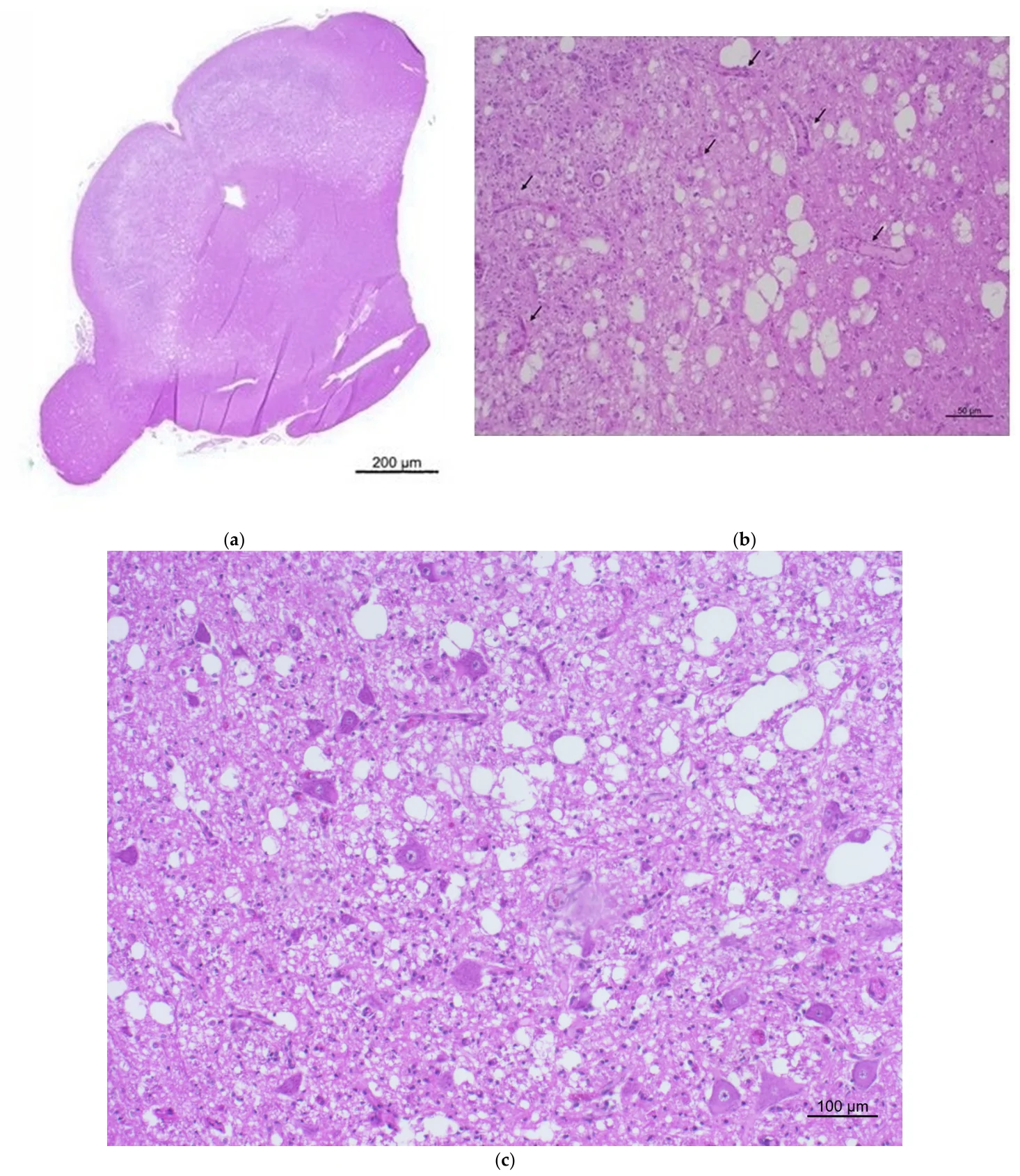
(**a**) Midbrain section characterized by bilateral and symmetrical vacuolation. H & E; 2.5×. (**b**) Midbrain showing an area of necrosis with spongiosis and capillary proliferation (black arrow). H & E; 20×. (**c**) Midbrain showing marked neuropil vacuolation and spongiosis, associated with tissue rarefaction, reactive gliosis, and relative preservation of neuronal cell bodies. H & E; 10×.

**Figure 3:**
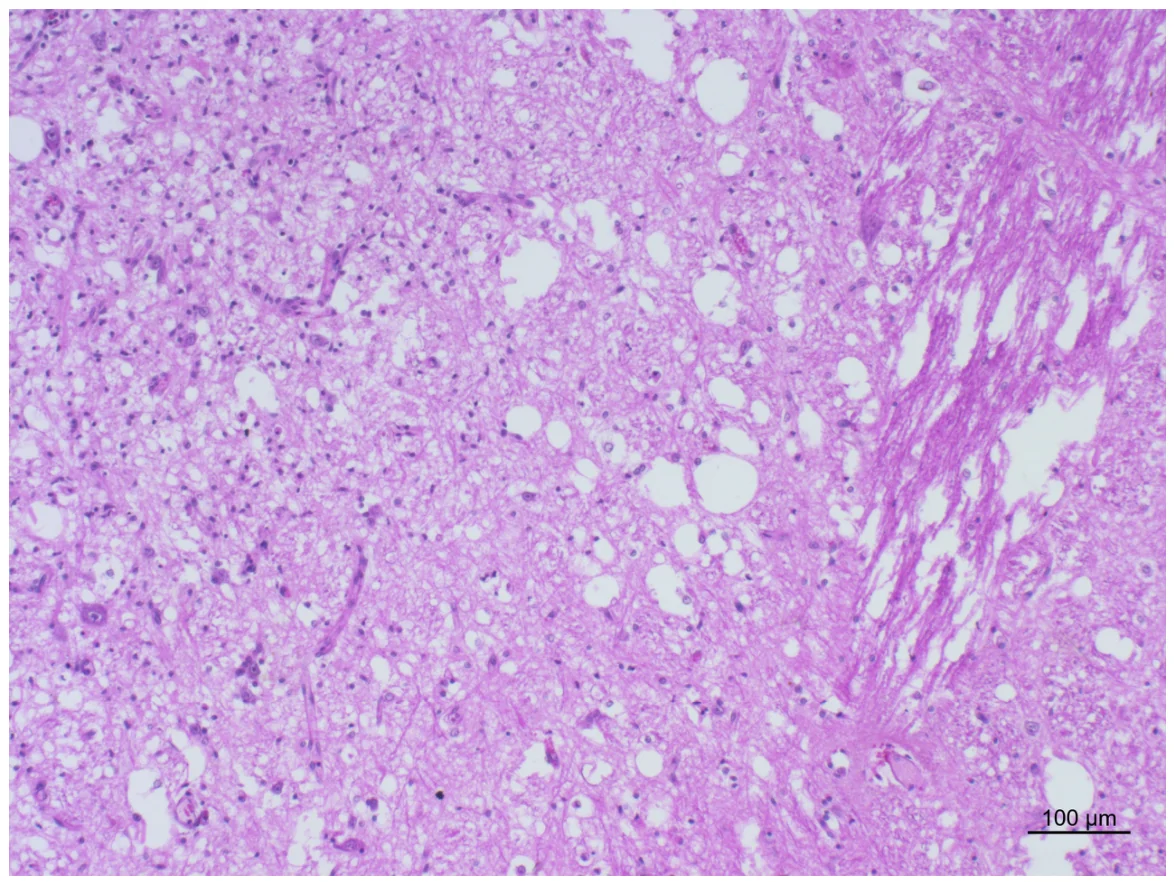
Pontine section showing marked spongiform degeneration of the grey matter, characterized by diffuse rarefaction and prominent vacuolation of the neuropil. H & E; 10×.

Gross examination revealed pulmonary and meningeal congestion together with pallor of the mucous membranes, findings consistent with respiratory failure as the cause of death. No macroscopic abnormalities were observed within the central nervous system (CNS), and the ventricular system appeared normal on sectioning. Histopathological examination of the CNS revealed destructive, multifocal, bilateral, and symmetrical lesions predominantly affecting several brain regions, with predominant involvement of the grey matter of the thalamus, midbrain, and pons, with no appreciable differences in lesion severity among these regions. The lesions were characterized by variable degrees of neuropil spongiosis and tissue necrosis/malacia (Figure 1, Figure 2 and Figure 3) associated with capillary proliferation (Figure 2b). Reactive gliosis and occasional perivascular lymphomonocytic aggregates were also observed (Figure 3). The affected areas showed axonal swelling and, where tissue destruction was most severe, relative preservation of neuronal cell bodies. The lesions were distributed bilaterally and symmetrically within the affected neuroanatomical regions.

**Figure 4:**
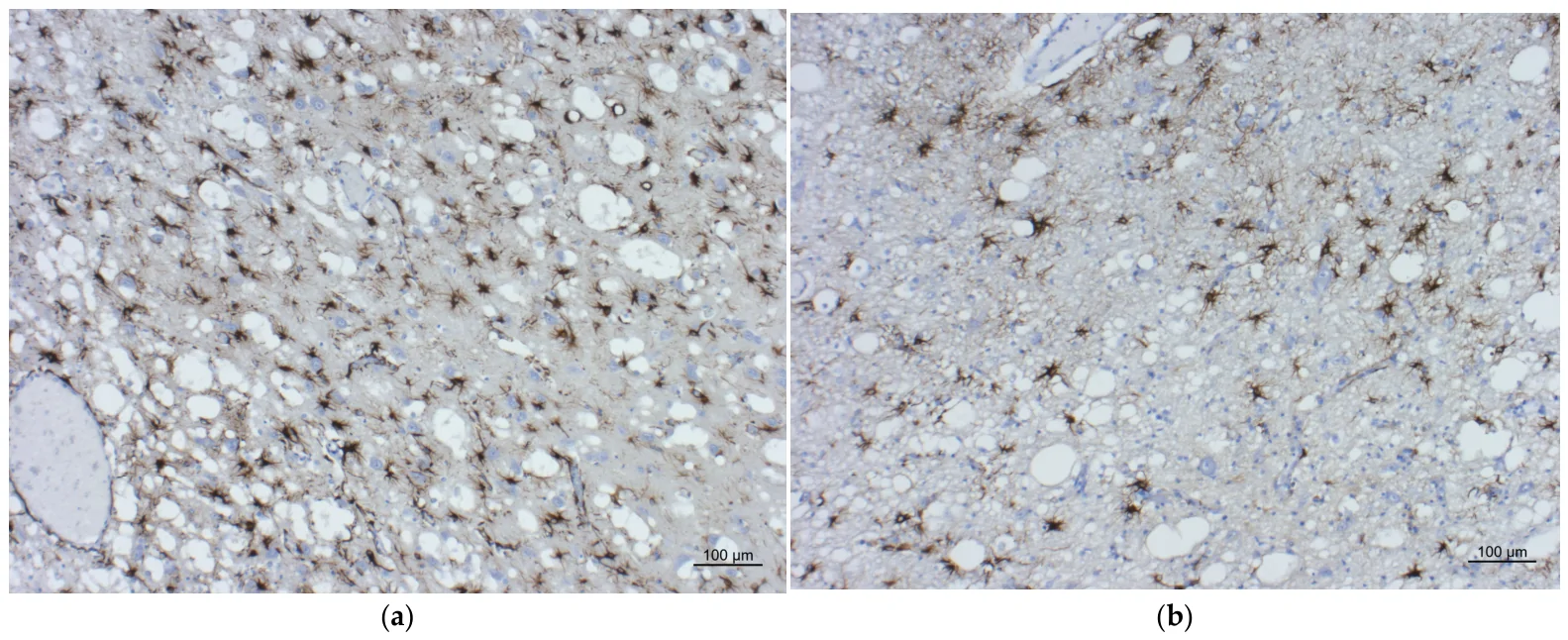
(**a**) Thalamus showing marked astrocytic gliosis, characterized by numerous GFAP-immunoreactive hypertrophic astrocytes with prominent cellular processes within the spongiotic area. GFAP IHC; 10×. (**b**) Midbrain showing marked astrocytic gliosis, characterized by numerous GFAP-immunoreactive hypertrophic astrocytes with prominent cellular processes within the spongiotic area. GFAP IHC; 10×.

FCoV IHC was negative, and no PrP^Sc^ accumulation was detected in any brain region. GFAP immunostaining demonstrated numerous reactive and hypertrophic gemistocytic astrocytes within the most severely affected areas, where bilateral and symmetrical spongiform lesions were observed (thalamus, midbrain, and pons) (Figure 4a,b).

Molecular analyses for FCoV, *Toxoplasma gondii*, FCV, and FHV-1 were negative, and both IF and virus isolation for FPV yielded negative results. Overall, the clinical presentation, lesion distribution, and neuropathological findings were consistent with a diagnosis of selective symmetrical encephalomalacia.

## 3. Discussion

The Maine Coon crossbred kitten described in this report showed neurological signs and neuropathological lesions consistent with SSE. A case of SSE in a two-year-old female cat has previously been described and differed from the present case because of the chronic, slowly progressive nature of the clinical signs, which progressed over 18 months [13]. In that report, degenerative, metabolic, or congenital disorders were considered the most likely differential diagnoses because of the chronic course and multifocal distribution of the lesions. In contrast, the kitten described here presented with rapidly progressive central vestibular signs similar to those reported in previous studies [2,6], followed by rapid clinical deterioration and natural death three days after the first veterinary examination. Consequently, inflammatory diseases of viral or protozoal origin, as well as idiopathic disorders, were also considered among the differential diagnoses.

Bilateral and symmetrical spongiform degeneration in young cats is not, by itself, specific for selective symmetrical encephalomalacia. Congenital and juvenile feline spongiform encephalopathies with partially overlapping clinical and neuroanatomical features have previously been described. Ref. [19] reported widespread vacuolation of both grey and white matter of the brain and spinal cord in two inbred littermate kittens, with ultrastructural evidence of predominantly intramyelinic vacuolation and expansion of the extracellular space, but without neuronal, axonal, or glial degeneration. Similarly, Jones et al. [20] described five related Birman kittens with diffuse spongiform changes and vacuolation of the brain and spinal cord associated with Wallerian degeneration, suggesting an inherited disorder. Other juvenile feline cases have shown spongiform changes predominantly affecting the grey matter or brainstem nuclei. Morita et al. [21] described a 9-month-old kitten with perineuronal and neuropil vacuolation caused predominantly by intramyelinic oedema, associated with mild-to-moderate astrocytosis but no neuronal loss, whereas Vidal et al. [22] reported generalized grey-matter vacuolation associated with reactive astrogliosis and ultrastructural swelling of neuronal and astrocytic processes. A 4-month-old Persian kitten described by Salvadori et al. [23] also showed multifocal, bilateral and symmetrical neuropil vacuolation involving several brainstem nuclei, but was characterized by prominent neuronal apoptosis.

In contrast, the present case was characterized not only by bilateral and symmetrical neuropil spongiosis, but by the concurrent presence of prominent tissue necrosis/malacia, capillary proliferation and vascular changes, and marked reactive gliosis, with relative preservation of neuronal cell bodies within the most severely affected areas. Moreover, the lesions showed a selective neuroanatomical distribution predominantly involving the grey matter of the thalamus, midbrain, and pons. This combination of destructive, vascular, and reactive changes distinguishes the present lesion pattern from previously reported congenital or juvenile feline spongiform encephalopathies in which vacuolation or intramyelinic oedema predominated without convincing evidence of malacia. In this respect, the neuropathological phenotype more closely resembles the selective symmetrical necrotizing encephalopathy previously reported by [13], in which spongiosis, necrotic/malacic lesions, reactive astrogliosis, and neovascularization were also documented. However, that case differed from the present one in its chronic clinical course and in the identification of mitochondrial genetic abnormalities. Taken together, these findings support interpretation of the present lesions as an SSE/Leigh-like neuropathological pattern rather than as a nonspecific form of feline spongiform degeneration.

Another cause of polioencephalomalacia in cats is thiamine deficiency, which may result from ingestion of food containing thiaminase, diets deficient in thiamine, or thiamine inactivation during food preservation [1].

Thiamine deficiency was considered less likely based on the dietary history and the distribution and morphology of the lesions, which differed from the bilateral, symmetrical hemorrhagic polioencephalomalacia previously described in a cat with thiamine deficiency [24]. However, in the absence of specific biochemical or other diagnostic testing, thiamine deficiency cannot be definitively excluded.

In humans, Leigh syndrome is characterized by bilateral, symmetrical necrotizing lesions predominantly affecting gray matter, whereas white matter lesions are typically associated with demyelination and spongiform degeneration [11]. Similar neuropathological findings, including neuronal loss and gliosis, have been described. In Alaskan Husky encephalopathy, the thalamus is the most severely affected region, showing bilateral and symmetrical cavitation, while involvement of the cerebral gray matter is also a consistent feature. Although differences in lesion topography exist between canine and human disorders, important similarities in clinical presentation have been reported [2]. Likewise, Collins et al. [6] described American Staffordshire Bull Terrier puppies affected by Leigh-like syndrome that presented with progressive neurological disease, central vestibular signs, and bilateral, symmetrical necrotizing encephalopathy. Neuropathological changes in those cases included marked neovascularization, gliosis, relative preservation of neuronal cell bodies, malacia, and vacuolation of the neuropil.

The case shared marked similarities with both Leigh syndrome and Alaskan Husky encephalopathy with respect to the kitten’s young age and the distribution and morphology of the neuropathological lesions. However, the clinical presentation more closely resembled that reported in American Staffordshire Bull Terrier puppies affected by Leigh-like syndrome [6].

Although the neuropathological phenotype observed in the present case shares important similarities with mitochondrial encephalopathies, a mitochondrial etiology cannot be considered established. Mitochondrial functional studies, respiratory chain analyses, ultrastructural investigations, and genetic testing were not performed in the present case. Therefore, mitochondrial disorder should be considered a possible underlying pathogenic mechanism rather than an established cause of the observed lesions.

In the present case, the classification of the lesions as an SSE/Leigh-like neuropathological pattern was based not only on their bilateral and symmetrical distribution but also on the combination of selective neuroanatomical involvement, neuropil spongiosis, tissue necrosis/malacia, vascular proliferation, and marked reactive gliosis. Relative preservation of neuronal cell bodies within areas of severe tissue destruction was also observed. These combined morphological features are considered more informative for identifying a Leigh-like/SSE pattern than bilateral symmetry alone.

Serum lactate concentrations have rarely been reported in Leigh-like canine disorders, with normal values described in Alaskan Huskies and increased values reported in 58% of affected American Staffordshire Bull Terrier puppies [6]. In the present case, cerebrospinal fluid was not available; therefore, only serum lactate concentration could be evaluated, and it was approximately twice the upper limit of the reference range. The timing and clinical conditions under which the blood sample was collected were not available; therefore, the relationship between the increased lactate concentration and the animal’s clinical status, including possible recent seizure activity or respiratory compromise, cannot be established. Accordingly, the increased serum lactate concentration should be interpreted cautiously and regarded as a supportive but nonspecific finding rather than evidence of primary mitochondrial dysfunction.

The negative immunohistochemical and molecular results for FCoV did not support a diagnosis of feline infectious peritonitis, despite the weakly positive antemortem test result. Furthermore, GFAP immunohistochemistry demonstrated numerous reactive and hypertrophic astrocytes within affected areas, in agreement with observations reported in Leigh syndrome [11], although the cytoplasmic vacuolation of astrocytes reported in previous studies [2,25] was not observed.

## 4. Conclusions

This report describes a rare case of selective symmetrical encephalomalacia (SSE) in a six-month-old Maine Coon crossbred kitten presenting with rapidly progressive neurological deterioration and a fatal outcome. The clinical and neuropathological findings showed important similarities with Leigh-like encephalopathies described in dogs and humans, particularly with regard to the selective bilateral and symmetrical distribution of lesions, spongiform change, tissue malacia, vascular proliferation, and reactive gliosis. Extensive histopathological, immunohistochemical, and molecular investigations did not provide evidence supporting the main infectious differential diagnoses considered.

The observed lesions are therefore compatible with an SSE/Leigh-like neuropathological phenotype. However, the underlying etiology remains undetermined. In particular, the increased serum lactate concentration and the morphological similarities with mitochondrial encephalopathies support mitochondrial disorder as a possible pathogenic mechanism, but do not demonstrate a primary mitochondrial disorder in the absence of specific functional, ultrastructural, or genetic investigations. This case contributes to the limited knowledge of SSE-like neuropathological lesions in cats and emphasizes the importance of detailed postmortem and neuropathological investigations in young animals with rapidly progressive neurological disease.

## Data Availability

The original contributions presented in this study are included in the article. Further inquiries can be directed to the corresponding author.

## Abbreviations

The following abbreviations are used in this manuscript:

SSE	selective symmetrical encephalomalacia
CNS	central nervous system
FIV	feline immunodeficiency virus
FeLV	feline leukemia virus
FCoV	feline coronavirus
FCV	feline calicivirus
FHV-1	feline herpesvirus type 1
FPV	feline panleukopenia virus
H & E	hematoxylin and eosin
IHC	immunohistochemistry
PrP^Sc^	disease-associated prion protein
GFAP	glial fibrillary acidic protein
ABC	avidin–biotin–peroxidase complex
IF	immunofluorescence
PCR	polymerase chain reaction
RT-PCR	reverse transcription polymerase chain reaction
ORF1	open reading frame 1
TK	thymidine kinase
EMEM	Eagle’s minimum essential medium
CRFK	Crandell–Rees feline kidney cells
UV	ultraviolet
